# Genome-Wide Analysis of the HSP20 Gene Family and Expression Patterns of *HSP20* Genes in Response to Abiotic Stresses in *Cynodon transvaalensis*

**DOI:** 10.3389/fgene.2021.732812

**Published:** 2021-09-08

**Authors:** Fengchao Cui, Geli Taier, Xiangfeng Wang, Kehua Wang

**Affiliations:** ^1^Department of Turfgrass Science and Engineering, College of Grassland Science and Technology, China Agricultural University, Beijing, China; ^2^National Maize Improvement Center, College of Agronomy and Biotechnology, China Agricultural University, Beijing, China

**Keywords:** *C. transvaalensis*, HSP20 gene family, gene duplication, *CtHSP20s*, heat stress

## Abstract

African bermudagrass (*Cynodon transvaalensis* Burtt–Davy) is an important warm-season turfgrass and forage grass species. Heat shock protein 20 (HSP20) is a diverse, ancient, and important protein family. To date, *HSP20* genes have not been characterized genome-widely in African bermudagrass. Here, we confirmed 41 *HSP20* genes in African bermudagrass genome. On the basis of the phylogenetic tree and cellular locations, the HSP20 proteins were classified into 12 subfamilies. Motif composition was consistent with the phylogeny. Moreover, we identified 15 pairs of paralogs containing nine pairs of tandem duplicates and six pairs of WGD/segmental duplicates of *HSP20* genes. Unsurprisingly, the syntenic genes revealed that African bermudagrass had a closer evolutionary relationship with monocots (maize and rice) than dicots (*Arabidopsis* and soybean). The expression patterns of *HSP20* genes were identified with the transcriptome data under abiotic stresses. According to the expression profiles, *HSP20* genes could be clustered into three groups (Groups I, II, and III). Group I was the largest, and these genes were up-regulated in response to heat stress as expected. In Group II, one monocot-specific *HSP20*, *CtHSP20-14* maintained higher expression levels under optimum temperature and low temperature, but not high temperature. Moreover, a pair of WGD/segmental duplicates *CtHSP20-9* and *CtHSP20-10* were among the most conserved *HSP20s* across different plant species, and they seemed to be positively selected in response to extreme temperatures during evolution. A total of 938 *cis*-elements were captured in the putative promoters of *HSP20* genes. Almost half of the *cis*-elements were stress responsive, indicating that the expression pattern of *HSP20* genes under abiotic stresses might be largely regulated by the *cis*-elements. Additionally, three-dimensional structure simulations and protein–protein interaction networks were incorporated to resolve the function mechanism of HSP20 proteins. In summary, the findings fulfilled the HSP20 family analysis and could provide useful information for further functional investigations of the specific *HSP20s* (e.g., *CtHSP20-9*, *CtHSP20-10*, and *CtHSP20-14*) in African bermudagrass.

## Introduction

As sessile organisms, plants are more prone to environmental stresses that could cause an adverse impact on growth and development, such as drought, salinity, cold, and high temperature (Filomena et al., 2009; Herman et al., 2017). For instance, to cope with the heat stress, plants regulate the gene expression patterns, resulting in a nearly exclusive synthesis of stress proteins, particularly heat shock proteins (HSPs; Sun et al., 2002). HSPs as molecular chaperones were distributed in diverse organisms and played crucial roles in assisting protein folding and preventing protein aggregation (Becker and Craig, 1994; Tyedmers et al., 2010). They are now also known to function in developmental stages and to respond to other abiotic stresses such as low temperature, drought, salinity, and stress-induced oxidative stress (Smirnoff, 1998; Yadav et al., 2021).

Generally, HSPs could be categorized into five protein families according to the molecular weight and sequence homology: HSP100s/ClpB, HSP90s, HSP70s/DnaK, HSP60s, and HSP20s (Wang et al., 2004; Waters, 2013). HSP20s are also called small HSP (sHSP), and its molecular weight is approximately between 12 and 42 kDa (Wang et al., 2004). Commonly, HSP20s capture the substrate proteins in an ATP-independent manner and prevent the irreversible aggregation of stress-denaturing proteins (Cashikar et al., 2005). The release and folding of the HSP20–substrate complexes are not spontaneous, and the ATP-dependent chaperones, including HSP70s and HSP100s, could cooperate in the processes (Lee et al., 1997; Haslbeck and Vierling, 2015). The HSP20 sequences contain the central conserved domain, the α-crystallin domain (ACD), which is flanked by a variable N-terminal region and a short C-terminal extension (Caspers et al., 1995; Kriehuber et al., 2010). The ACD is the signature domain of HSP20s containing 80–100 amino acids, and its structure in plants is a β-sandwich including three and four strands in an antiparallel direction and an extended strand (β6; Scharf et al., 2001; Basha et al., 2012). Unlike other HSP families, the HSP20 family is more variable and diverged in plant kingdom (Basha et al., 2012). On the basis of subcellular locations, seven subfamilies (CI, CII, CIII, M, P, ER, and Px) were primarily defined in *Arabidopsis* (Scharf et al., 2001). Another five subfamilies, including four cytoplasmic subfamilies (CIV, CV, CVI, and CVII) and one mitochondrial subfamily MII (Siddique et al., 2008), were added into the former classification. Totally, HSP20 proteins could be classified into 12 subfamilies (CI, CII, CIII, CIV, CV, CVI, CVII, MI, MII, ER, P, and Po; Scharf et al., 2001; Ma et al., 2006; Siddique et al., 2008). Among the subfamilies, CI to CVII are localized to the cytoplasmic/nuclear, M (MI and MII) are localized to the mitochondria, and ER, P, and Po are localized to the endoplasmic reticulum (ER), plastids (Ps), and peroxisomes (Pos), respectively.

In recent years, as more plant genomes have been assembled, the HSP20 gene family was identified across many plant species including *Arabidopsis* (*Arabidopsis thaliana*; Siddique et al., 2008), rice (*Oryza sativa*; Ouyang et al., 2009), soybean (*Glycine max*; Lopes-Caitar et al., 2013), wheat (*Triticum aestivum*; Muthusamy et al., 2017), potato (*Solanum tuberosum*; Zhao et al., 2018), and apple (*Malus domestica*; Yao et al., 2020). Of the species, there are 19 *HSP20* genes in *Arabidopsis*, 39 in rice, 51 in soybean, 163 in wheat, 48 in potato, and 41 in apple. *Cynodon transvaalensis* Burtt–Davy, commonly called African bermudagrass, is a perennial warm-season turfgrass species. Recently, the genome of African bermudagrass was assembled (Cui et al., 2021), which makes it possible to characterize the HSP20 gene family in African bermudagrass genome-widely. African bermudagrass is primarily endemic to damp and uncultivated areas including Orange Free State, southern Transvaal and northern Cape Province of South Africa, and was further introduced to other countries such as Greece, Iran, the United States, Madagascar, and Australia (Beard, 2013). Although the commercial use of African bermudagrass for turf was relatively limited, African bermudagrass exhibits a great value as a parent in hybridization with tetraploid *C. dactylon* and has been utilized to breed a lot of leading triploid bermudagrass cultivars.

Here we utilized bioinformatics methods to identify *HSP20* genes of African bermudagrass and uncovered their chromosomal positions, gene duplication events, and phylogenetic relationships. Moreover, the expression patterns of *HSP20* genes were analyzed with RNA-seq data to determine their responses to abiotic stresses including drought, salinity, and extreme temperatures. The protein three-dimensional (3D) structures and their protein–protein interaction (PPI) networks were all predicted to resolve the possible regulation mechanisms of HSP20s. The findings in the study would provide valuable information for further investigations of the functions and regulatory mechanisms of potentially important *HSP20* genes in modulating African bermudagrass tolerance to abiotic stresses, including high temperature.

## Materials and Methods

### Identification and Characterization of Heat Shock Protein 20 Family Members in African Bermudagrass Genome

To identify HSP20 candidates, the HSP20 Hidden Markov model (HMM) profile (PF00011) was downloaded from Pfam^1^, and the HMM was used to screen the whole genome of African bermudagrass (Cui et al., 2021). The candidate proteins were found with software HMMER v3.2.1^2^. To avoid missing possible candidates, a *C. transvaalensis-*specific HMM was constructed based on high-quality domain sequences (*E*-value < 1e-20). The second searching was preformed, and the outputs were combined with previous results with *E*-value 0.01. The non-redundant putative HSP20 proteins were confirmed with Pfam, NCBI Conserved Domain Database^3^, and SMART^4^ (Ivica and Peer, 2018). Additionally, the protein theoretical isoelectric points (pI) and molecular weights (MW) were estimated with ExPASy^5^. The chromosomal positions of high-confidence *HSP20* genes were visualized using TBtools (Chen et al., 2020).

### Gene Duplication and Non-synonymous (Ka) and Synonymous (Ks) Calculation

In *CtHSP20s* of African bermudagrass, gene duplications were identified with MCScanX (Wang et al., 2012) using BLASTP results (*E*-value < 1e-10; Camacho et al., 2009). In MCScanX program, all genes were relabeled with gene ranks according to their chromosomal positions. If the rank difference of BLASTP hits was equal to 1, the two genes were “tandem duplicates.” For the genes anchored in colinear blocks, they were classified into “WGD/segmental duplicates.” If genes had multiple BLASTP hits, WGD/segmental duplicates had higher priority than tandem duplicates. The tandem and WGD/segmental duplicated events were visualized with Circos^6^ (Krzywinski et al., 2009). In addition, the HSP20 syntenic blocks among African bermudagrass and other plant genomes (*Arabidopsis*, soybean, maize, and rice) were detected (cscore ≥ 0.70) and displayed with MCscan^7^ (Tang et al., 2008). OrthoFinder (Emms and Kelly, 2019) was utilized to infer orthologous genes among African bermudagrass and other representative species. The model-averaged method was adopted to estimate non-synonymous (Ka), synonymous (Ks) values, and Ka/Ks ratios with KaKs_Calculator 2.0 (Wang et al., 2010). Commonly, neutral mutation was defined as Ka/Ks = 1, and Ka/Ks > 1 and Ka/Ks < 1 represented positive and negative (purifying) selection, respectively (Wang et al., 2010).

### Phylogenetic Analysis

Multiple alignments of HSP20 full-length amino acid sequences derived from African bermudagrass, *Arabidopsis*, rice, maize, and soybean were performed with ClustalW (Thompson et al., 1994). The poorly aligned regions were trimmed manually, and the unrooted phylogenetic trees were estimated by neighbor-joining method with MEGA X^8^ (Sudhir et al., 2018) using the following parameters: Poisson model, pairwise deletion, and 1,000 bootstrap replicates. Additionally, among the African bermudagrass HSP20 proteins, the phylogenetic tree was also constructed with the method above, and the MEME program^9^ was utilized to identify conserved motifs with a maximum of 10 motifs and a width of 5–50 amino acids. The two phylogenetic trees were both polished with ITOL^10^.

### Plant Treatment and Expression Analysis of *HSP20* Genes

The Illumina RNA-seq data of African bermudagrass shoot for various treatments, including optimum temperature (RTS, 25/30°C, day/night, and control), drought stress (DSS, water withholding for 5 days with a relative leaf water content of ∼60%), salinity stress (SSS, 200 mM NaCl for 24 h, soil salinity was increased by 50 mM daily), high temperature (HTS, 45°C for 6 h), and low temperature stress (LTS, 4°C for 6 h), were generated by our lab recently and accessible under BioProject PRJCA003581 of the China National Center for Bioinformation GSA (Genome Sequence Archive) database, and the data were utilized to explore the expression patterns and cluster expression groups of *CtHSP20s*. Transcripts per million (TPM) of *HSP20* genes across different environments were transformed with z-score.

### *Cis*-Element Analysis of HSP20 Gene Promoters

The 1.5-kb upstream sequences of *HSP20* genes were extracted as putative promoters, and these sequences were submitted to PlantCARE^11^ (Lescot et al., 2002) to analyze *cis*-regulatory elements. The heat shock-responsive elements (HSEs) were predicted with FIMO^12^ (Noble, 2011), a part of the MEME software toolkit, using the sequence module nGAAnnTTCnnGAAn or nTTCnnGAAnnTTCn (Sarkar et al., 2009; Lopes-Caitar et al., 2013). Two-tailed Fisher’s exact test was used to examine the relationship between HSEs and up-regulated *HSP20* genes. Total *cis*-elements in promoter sequences were plotted with TBtools (Chen et al., 2020).

### Three-Dimensional Protein Structure Prediction

The 3D structures of HSP20 proteins were predicted with SWISS-MODEL^13^, which was a fully automated sever dedicated to protein structure homology modeling. In addition, the model quality was evaluated by global model quality estimation (GMQE) and QMEAN. The GMQE score is between 0 and 1, and the higher score indicates the model is more reliable. The QMEAN score around 0 indicates high quality, and -4 or below indicates low quality.

### Protein–Protein Interaction Network

To predict the relationships among HSP20 proteins and other related proteins, HSP20 protein sequences were submitted to STRING v11.0 database^14^ (Damian et al., 2018). The organism was set to rice, and the advanced settings were kept in default mode. The PPI networks were visualized with Cytoscape v3.7.2 (Shannon et al., 2003).

## Results

### Identification, Characterization, and Distribution of HSP20 Family Genes in African Bermudagrass

On the basis of the newly assembled African bermudagrass genome (Cui et al., 2021), the HSP20 candidate sequences were identified with the HSP20 HMM (PF00011). The *C. transvaalensis*-specific HMM was built with domains (*E*-value < 1e-20). A total of 45 sequences were detected under the threshold *E*-value < 0.01. In addition, the sequences were confirmed with the ACD domain using Pfam, CDD, and SMART (Ivica and Peer, 2018). The sequence molecular weight not in the range of 12–42 kDa was excluded. Finally, the remaining 41 high-confidence candidates were preserved as the HSP20 members and were named with CtHSP20-1 to CtHSP20-41 according to their chromosomal positions.

The characteristics of the HSP20 members, containing gene names, gene IDs, chromosomal locations, open reading frame lengths, amino acid numbers, MW, and isoelectric points, were summarized in Table 1. Overall, the amino acid numbers were from 117 (CtHSP20-29) to 328 (CtHSP20-6). The predicted MW of the HSP20 proteins were between 13.20 (CtHSP20-29) and 36.62 kDa (CtHSP20-6), and the predicted isoelectric points varied from 4.90 (CtHSP20-35) to 9.76 (CtHSP20-25).

**TABLE 1:** The characteristics of heat shock protein 20 (HSP20) members identified in African bermudagrass.

Gene name	Gene ID	Chr	Genomic locations	CDS (bp)	AA	MW (kDa)	pI
CtHSP20-1	evm.model.LG01.398	Chr1	2,831,464–2,832,277	720	239	25.66	5.88
CtHSP20-2	evm.model.LG01.916	Chr1	6,892,913–6,893,731	720	239	26.70	6.02
CtHSP20-3	evm.model.LG01.1036	Chr1	7,891,976–7,892,755	654	217	24.40	7.98
CtHSP20-4	evm.model.LG01.1038	Chr1	7,909,811–7,910,282	471	156	17.65	6.19
CtHSP20-5	evm.model.LG01.1039	Chr1	7,910,881–7,911,343	462	153	17.46	6.19
CtHSP20-6	evm.model.LG01.2241	Chr1	23,349,398–23,350,475	987	328	36.62	9.35
CtHSP20-7	evm.model.LG02.592	Chr2	11,068,319–11,068,933	531	176	19.47	9.27
CtHSP20-8	evm.model.LG02.965	Chr2	18,824,494–18,841,582	768	255	27.84	5.90
CtHSP20-9	evm.model.LG02.1296	Chr2	24,091,622–24,092,237	480	159	17.84	5.60
CtHSP20-10	evm.model.LG02.1517	Chr2	27,953,172–27,953,787	480	159	17.76	5.82
CtHSP20-11	evm.model.LG02.2172	Chr2	34,141,399–34,141,858	459	152	16.93	6.18
CtHSP20-12	evm.model.LG03.433	Chr3	3,764,103–3,764,778	558	185	21.25	9.36
CtHSP20-13	evm.model.LG03.713	Chr3	6,530,821–6,531,268	447	148	16.47	6.85
CtHSP20-14	evm.model.LG03.714	Chr3	6,538,664–6,539,126	462	153	16.60	4.98
CtHSP20-15	evm.model.LG03.715	Chr3	6,545,759–6,546,215	456	151	17.05	5.79
CtHSP20-16	evm.model.LG03.716	Chr3	6,547,591–6,548,050	459	152	17.08	6.76
CtHSP20-17	evm.model.LG03.994	Chr3	9,259,177–9,259,663	486	161	17.76	5.97
CtHSP20-18	evm.model.LG03.3139	Chr3	43,298,792–43,299,428	504	167	17.67	8.95
CtHSP20-19	evm.model.LG03.3140	Chr3	43,302,943–43,303,924	981	326	35.64	9.08
CtHSP20-20	evm.model.LG04.203	Chr4	1,794,764–1,795,298	534	177	19.62	6.85
CtHSP20-21	evm.model.LG04.595	Chr4	5,022,583–5,023,333	660	219	23.73	6.35
CtHSP20-22	evm.model.LG04.659	Chr4	5,719,610–5,720,150	540	179	19.66	5.41
CtHSP20-23	evm.model.LG04.1562	Chr4	15,238,517–15,239,382	537	178	19.64	9.33
CtHSP20-24	evm.model.LG04.1746	Chr4	18,664,184–18,664,932	636	211	23.30	8.98
CtHSP20-25	evm.model.LG04.1747	Chr4	18,667,791–18,669,012	633	210	22.63	9.76
CtHSP20-26	evm.model.LG04.2200	Chr4	27,791,470–27,792,269	696	231	25.21	9.65
CtHSP20-27	evm.model.LG04.2202	Chr4	27,841,964–27,842,642	555	184	20.52	6.31
CtHSP20-28	evm.model.LG04.3547	Chr4	40,383,326–40,383,791	465	154	17.27	6.44
CtHSP20-29	evm.model.LG04.3818	Chr4	42,785,564–42,785,918	354	117	13.20	5.30
CtHSP20-30	evm.model.LG04.3952	Chr4	43,822,911–43,823,827	504	167	18.15	6.66
CtHSP20-31	evm.model.LG05.1343	Chr5	44,933,904–44,934,507	603	200	21.45	6.01
CtHSP20-32	evm.model.LG05.1634	Chr5	51,747,665–51,786,045	444	147	15.45	5.89
CtHSP20-33	evm.model.LG06.1594	Chr6	12,730,245–12,730,890	645	214	23.52	5.74
CtHSP20-34	evm.model.LG08.943	Chr8	15,904,441–15,905,347	750	249	27.53	8.95
CtHSP20-35	evm.model.LG08.1754	Chr8	24,644,033–24,645,368	585	194	21.73	4.90
CtHSP20-36	evm.model.LG08.2303	Chr8	29,138,893–29,139,831	828	275	30.77	9.39
CtHSP20-37	evm.model.LG09.715	Chr9	7,378,771–7,379,530	633	210	22.79	5.69
CtHSP20-38	evm.model.LG09.716	Chr9	7,401,085–7,401,854	633	210	23.17	5.61
CtHSP20-39	evm.model.LG09.717	Chr9	7,406,158–7,406,950	660	219	23.86	5.87
CtHSP20-40	evm.model.LG09.718	Chr9	7,409,020–7,409,923	774	257	27.67	5.66
CtHSP20-41	evm.model.LG09.852	Chr9	8,975,686–8,976,118	432	143	15.68	8.05

**FIGURE 1:**
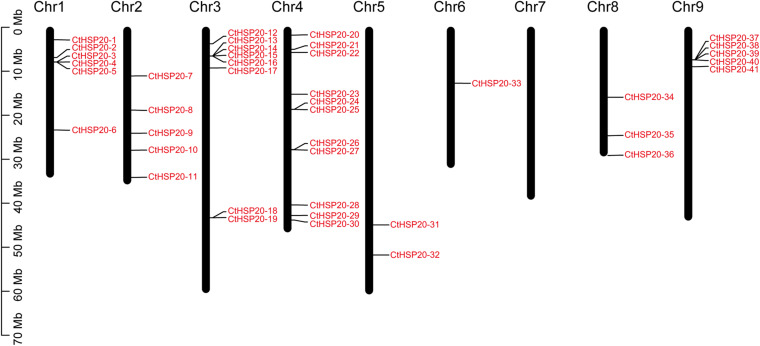
The chromosomal locations of the heat shock protein 20 (HSP20) members. The long black bars represent the chromosomes. The chromosome numbers are labeled on the top of the bars, and the red fonts represent the HSP20 members.

The *CtHSP20s* were unevenly distributed across the eight of nine chromosomes in African bermudagrass (Figure 1). As shown in Figure 1, most *HSP20* genes were located on the first four chromosomes (Chr1 to Chr4). Chr4 contained the most *HSP20* genes (11), although it was not the longest chromosome. Gene clusters could be observed on Chr1, Chr3 and Chr4, and Chr9. We also noted that there was no *HSP20* genes on Chr7, and relatively less *HSP20* genes were located on Chr5, Chr6, and Chr8.

### Syntenic Gene Analysis of *HSP20* Genes in African Bermudagrass

**FIGURE 2:**
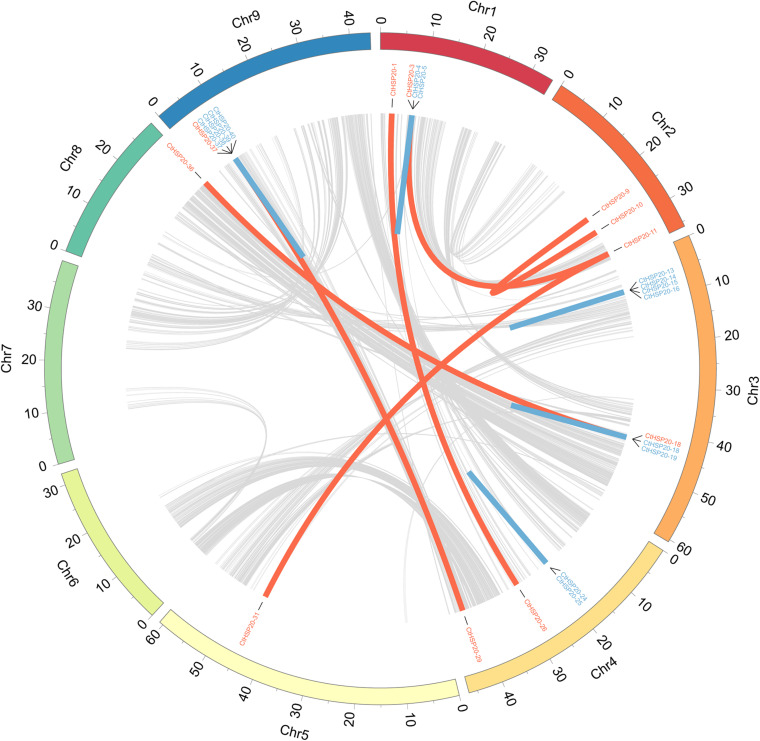
Synteny patterns and gene duplications of African bermudagrass HSP20 gene family. Tandem and WGD/segmental duplicates are exhibited with blue and red lines, respectively. Interchromosomal synteny blocks are exhibited with gray lines.

To investigate the gene duplication events, synteny analysis was conducted to the *HSP20* genes using BLASTP (Camacho et al., 2009) and MCScanX (Wang et al., 2012). Totally, there were 15 pairs of paralogous genes among *HSP20* genes (Figure 2). In the paralogs, nine pairs of genes were identified as tandem duplicates distributed as clusters on Chr1, Chr3, Chr4, and Chr9, respectively. Besides, six pairs of genes were defined as WGD/segmental duplicates, in which one pair (CtHSP20-9 vs. CtHSP20-10) located on Chr2 was intrachromosomal, and the other pairs of genes were inter-chromosomal (Supplementary Table 1). As a result, tandem and WGD/segmental duplicates were both important components in the HSP20 gene family.

**FIGURE 3:**
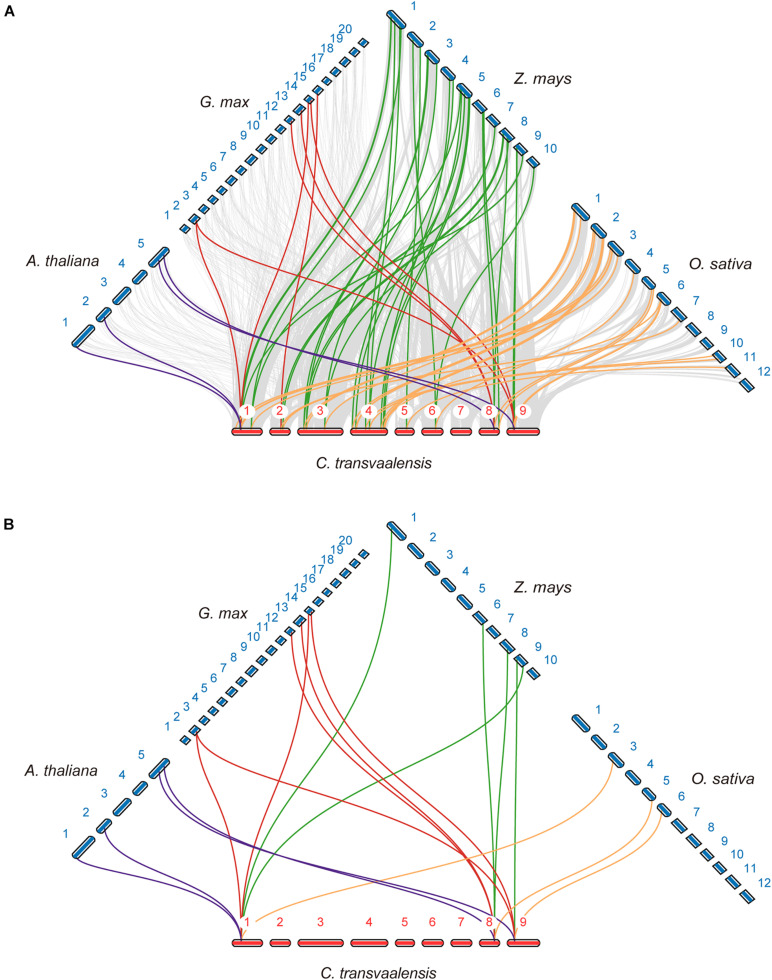
Synteny analysis of *CtHSP20s* between African bermudagrass and other species. **(A)** Syntenic genes of *CtHSP20s* among African bermudagrass and *Arabidopsis* (*A. thaliana*), *Glycine max* (*G. max*), *Zea mays* (*Z. mays*), and *Oryza sativa* (*O. sativa*) are exhibited with purple, red, green, and yellow lines, respectively. Gray lines indicate the synteny blocks. **(B)** Three *CtHSP20s* (CtHSP20-3, CtHSP20-35, and CtHSP20-41) having syntenic genes among four species were exhibited with purple, red, green, and yellow lines as well.

To further explore the gene duplication of *CtHSP20s*, a comparative analysis was conducted to four representative species, containing two monocots (maize and rice) and two dicots (soybean and *Arabidopsis*). Thirty-three and 26 *CtHSP20s* syntenic genes were identified in maize and rice, respectively, followed by *Arabidopsis* (4) and soybean (7; Figure 3A). Three *CtHSP20s* (CtHSP20-3, CtHSP20-35, and CtHSP20-41) existed syntenic genes across the four species (Figure 3B). In addition to syntenic block analysis, we also separately detected *CtHSP20s* orthologs in *Arabidopsis*, soybean, rice, and maize with OrthoFinder (Emms and Kelly, 2019). Both rice and maize contained orthologs of 36 *CtHSP20s*, and *Arabidopsis* and soybean had orthologs of 29 and 31 *CtHSP20s*, respectively. All the 41 *CtHSP20s* had orthologous genes in at least one of the four species, and 23 of them had orthologous genes among all the four species. As anticipated, the functions of most *CtHSP20* orthologous genes are stress related (heat responsive, oxidative responsive, hypoxia responsive, etc.; Supplementary Table 2).

In order to investigate the evolutionary dynamics for *CtHSP20s* protein-coding sequences, non-synonymous (Ka), synonymous (Ks) substitution rates, and Ka/Ks ratios were calculated. The comprehensive information is listed in Supplementary Table 3. Among *CtHSP20s* paralogs, only one pair of WGD/segmental duplicate (CtHSP20-9 vs. CtHSP20-10) was positively selected (Ka/Ks > 1), and the remaining pairs of genes might experience purifying selection (Ka/Ks < 1; Supplementary Table 3). Among different species, the syntenic gene pairs might experience purifying selection (Ka/Ks < 1) as well (Supplementary Table 4).

### Phylogenetic and Motif Analyses of the Heat Shock Protein 20 Proteins

To further uncover the evolutionary relationships of the HSP20 gene family, the unrooted phylogenetic tree was constructed with full-length amino acid sequences from multiple species, including *Arabidopsis*, rice, soybean, maize, and African bermudagrass. The identified HSP20 proteins were combined with previous studies (Muthusamy et al., 2017; Zhao et al., 2018), and a total of 174 protein sequences were used for multiple alignments. After removing two diverged sequences (Zm00001d014149 and Zm00001d037633), 172 sequences including 19 from *Arabidopsis*, 22 from rice, 44 from soybean, 46 from maize, and 41 sequences from African bermudagrass were preserved for further analyses.

**FIGURE 4:**
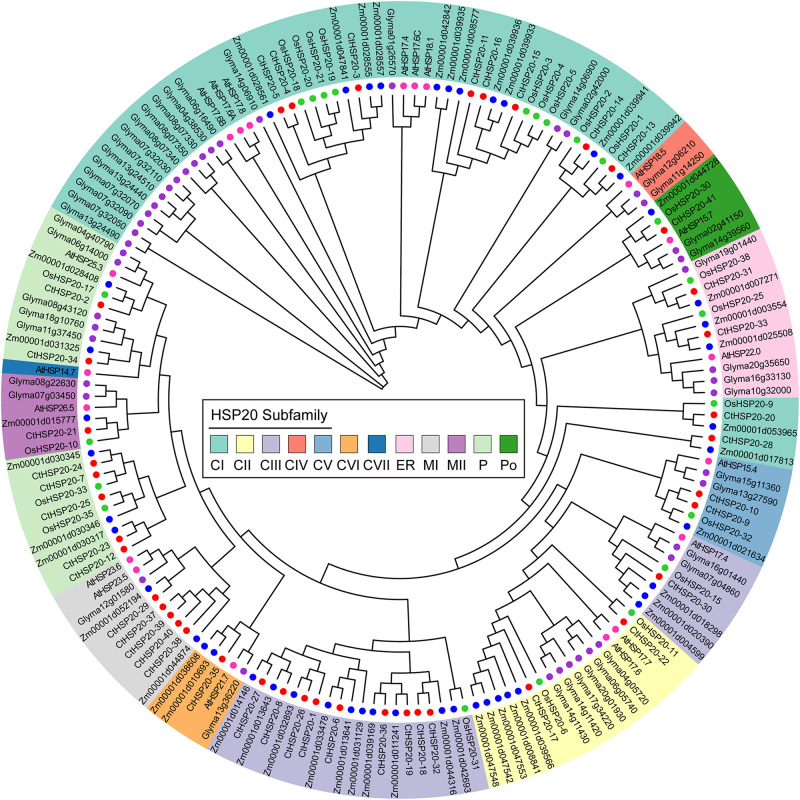
The phylogenetic relationships of the HSP20 proteins from African bermudagrass, *Arabidopsis*, rice, soybean, and maize. The neighbor-joining tree (bootstrap value = 1,000) was constructed with MEGA X. Twelve subfamilies are shaded with different colors, and the colored circles next to the tree branches represent different species.

Combining the phylogeny analysis and previously reported results (Scharf et al., 2001; Siddique et al., 2008; Muthusamy et al., 2017; Zhao et al., 2018), the HSP20 proteins were categorized into 12 distinct subfamilies, 56 cytosol Is (CIs), 17 CIIs, 28 CIIIs, 3 CIVs, 7 CVs, 5 CVIs, 1 CVIIs, 10 mitochondria Is (MIs), 6 mitochondria IIs (MIIs), 21 Ps, 6 Pos, and 12 ER (Figure 4). A total of 117 HSP20 proteins were classified into CI–CVII subfamilies, among which CIs was the largest subfamily containing 56 members, whereas CVII subfamily was the smallest containing only one *Arabidopsis* protein. The HSP20s of M subfamilies (MI and MII) were close to those of P subfamily in the phylogenetic tree, which was similar to soybean HSP20 results (Lopes-Caitar et al., 2013). Consistent with previous results (Siddique et al., 2008; Zhao et al., 2018; Yao et al., 2020), the CIV subfamily of our phylogenetic tree only contained HSP20 proteins of dicot plants as well.

**FIGURE 5:**
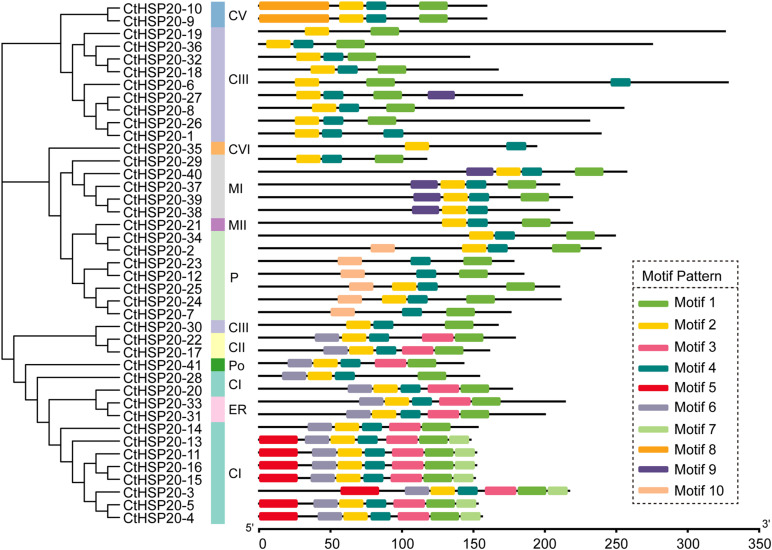
The phylogenetic relationships and domain analyses of HSP20 proteins in African bermudagrass. The phylogenetic tree is listed on the left, and the motifs are exhibited on the right. The bottom bar indicates HSP20 protein lengths.

To resolve the motif composition of *CtHSP20s*, the 41 sequences were submitted to the MEME website, and a total of 10 motifs were predicted (Figure 5). The length of the motifs ranged from 15 to 50 amino acids, and detailed motif sequences are provided in Supplementary Table 5. Among the 10 motifs, Motif 1, Motif 2, and Motif 4 were widespread on all the *CtHSP20s*. Subfamily CIII tended to contain a minimum of two to three motifs, whereas some sequences in subfamily CI had a maximum of seven motifs. Motif 8 was specific to subfamily CV, and Motif 9 was mainly contained in subfamily MI except one in subfamily CIII. Motif 5 and Motif 10 were unique to subfamily CI and P, respectively. Motif 3 and Motif 6 were specific to the bottom cluster according to the phylogenetic tree. Overall, the *CtHSP20s* motifs shared similar patterns with the phylogeny and the categorized HSP20 subfamilies. However, more analyses will be needed to elucidate the motif functions in the future.

### *HSP20* Gene Expression Profiles in Response to Multiple Abiotic Stresses

**FIGURE 6:**
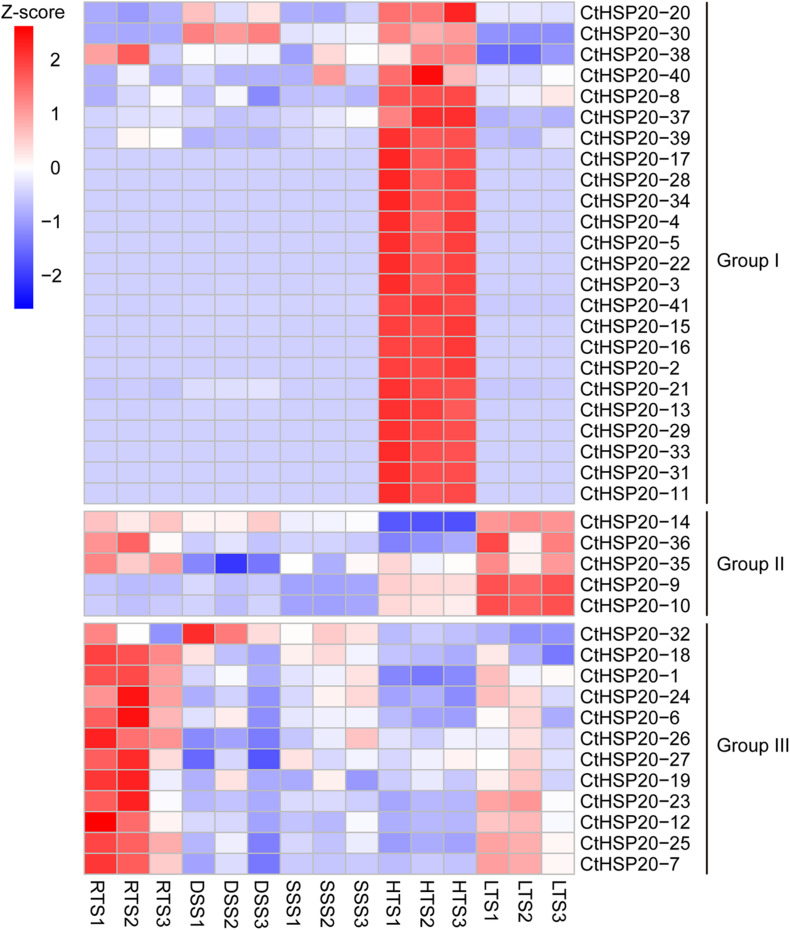
Expression profiles of *HSP20* genes in African bermudagrass under multiple abiotic stresses. *Z*-score transformed TPM values from transcriptome data were clustered. RTS, DSS, SSS, HTS, and LTS represent optimum temperature, drought stress, salinity stress, high temperature, and low temperature stress, respectively.

The *CtHSP20s* gene expression patterns were investigated to evaluate whether these genes were functional across multiple abiotic stresses containing RTS, DSS, SSS, HTS, and LTS. Each treatment had three biological replicates. The relative expression levels were represented by TPM values, which were calculated with transcriptome data generated by our lab before. All *CtHSP20s* genes were expressed (TPM > 0) in at least one treatment. Interestingly, most *CtHSP20s* genes were up-regulated in RTS, HTS and LTS treatments, and the up-regulated genes did not belong to the same set. Nevertheless, only several genes were expressed in DSS and SSS treatments. According to the relative expression profiles, *CtHSP20s* genes could be classified into three groups (Groups I, II, and III; Figure 6). Group I was the largest containing 24 members. Most genes of Group I were only up-regulated under HTS. Besides, CtHSP20-30 and CtHSP20-38 are also expressed in RTS and DSS. Group II only included five members, which were highly expressed in LTS, and three of them were also expressed in RTS. Most genes of Group III were up-regulated in RTS and down-regulated in other treatments.

### *Cis*-Element Analysis of HSP20 Gene Promoters

**FIGURE 7:**
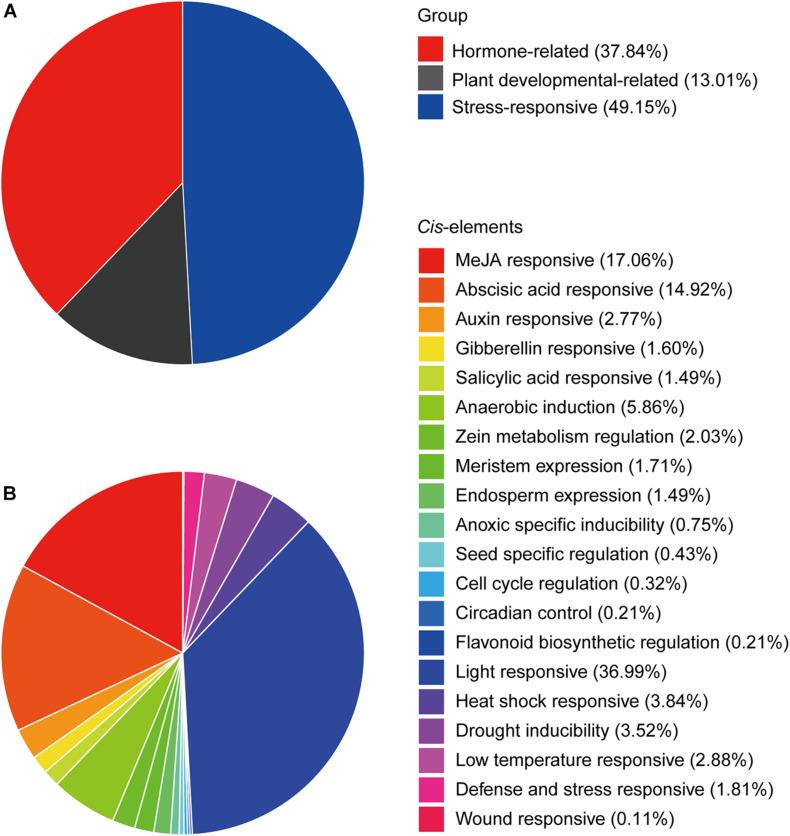
Statistical summary of *cis*-elements. **(A)** The percentage of three kinds of *cis*-elements. **(B)** The detailed percentages of each type of *cis*-element.

**FIGURE 8:**
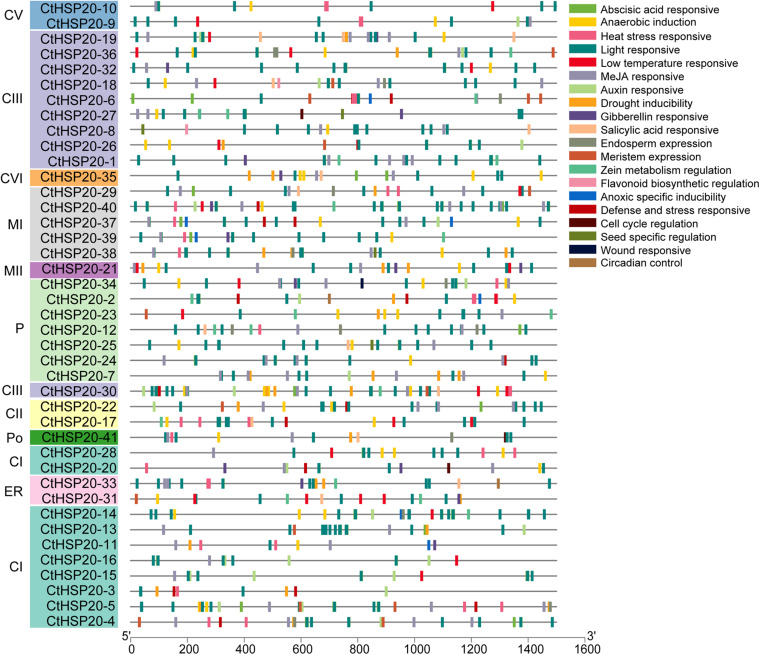
*Cis*-element distributions in putative promoters of *HSP20* genes in African bermudagrass. The bottom bar indicates the putative promoter lengths of *HSP20* genes.

To identify the potential roles of *cis*-elements, the promoter sequences, 1.5-kb upstream of the *CtHSP20s* genes, were extracted and submitted to PlantCARE (Lescot et al., 2002) and FIMO (Noble, 2011) to predict *cis*-acting elements. After removing non-functional terms, a total of 938 *cis*-elements could be classified into stress-responsive elements (heat stress, light, drought, wound, low temperature, and defense and stress), hormone-related elements (MeJA, abscisic acid, auxin, gibberellin, and salicylic acid), and plant development-related elements (meristem expression, cell cycle, and circadian control, etc.; Figures 7, 8 and Supplementary Table 6). Stress-responsive elements accounted for the largest proportion (49.15%), followed by hormone-related elements (37.84%) and plant development-related elements (13.01%; Figure 7A). As shown in Figure 7B, light-responsive elements were the most abundant, accounting for 36.99% of all elements, and it contained 26 kinds of motifs. Notably, HSEs (accounting for 3.84%) were distributed across 21 *CtHSP20s* genes, most of which were located in the Group I (Figure 6). Thirty-three and 27 *cis*-elements were also found to be drought and low temperature responsive, respectively. Among hormone-related elements, MeJA responsive (TCACG-motif and CGTCA-motif), abscisic acid responsive, auxin responsive (AuxRR-core and TGA-element), gibberellin responsive (P-box, TATC-box and GARE-motif), and salicylic acid responsive (TCA-element) motifs were widely distributed in *CtHSP20s* promoters. Less *cis*-acting elements (13.01%) were predicted in different developmental processes of African bermudagrass. In conclusion, the ubiquitous *cis*-acting elements could be involved in *CtHSP20s* gene expression regulation in response to multiple abiotic stresses.

### Three-Dimensional Structure Prediction and Protein–Protein Interaction Network

**FIGURE 9:**
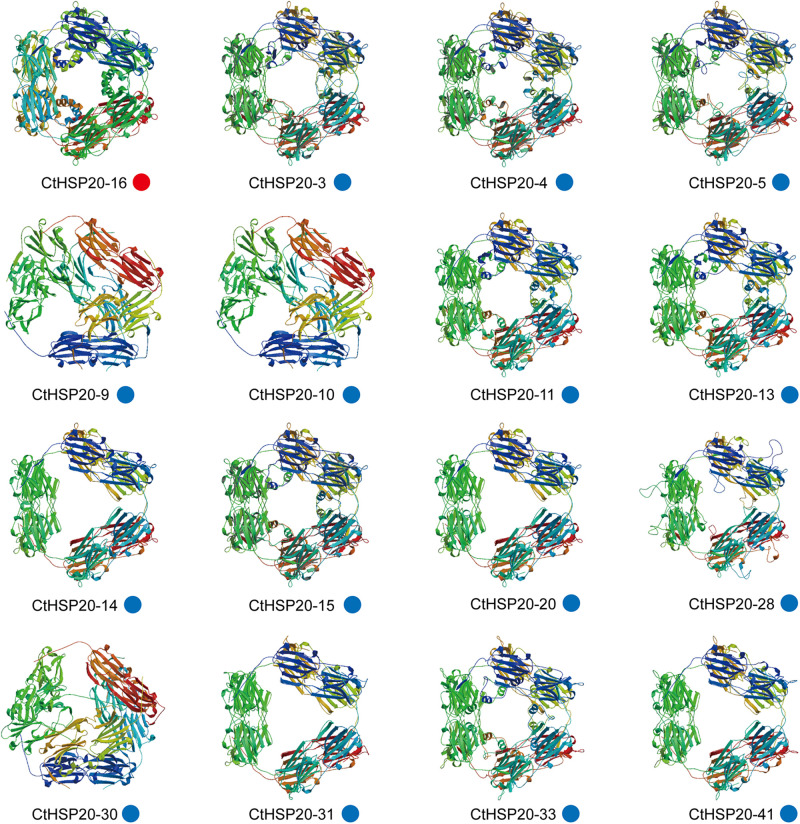
Three-dimensional (3D) protein structures of *CtHSP20s*. Broad strips are β-sheets, spirals are α-helices, and thin loops are coils. The colored circles at the bottom indicate different homology modeling templates. The red and blue circles indicate homology modeling templates 1gme.1.A and 1gme.2.A, respectively.

Three-dimensional protein structures of *CtHSP20s* were predicted with SWISS-MODEL (see text footnote 13). Subsequently, 23 successful models were defined by at least 30% identity of target to template, which was a widely accepted threshold for successful modeling (Xiang, 2006). Most QMEAN *z*-score values varies from -3.92 to 0.83, whereas QMEAN of CtHSP20-34 and CtHSP20-40 were -6.34 and -4.73, respectively, indicating that both models were of low quality. The GMQE values ranged from 0.19 to 0.75. Among 23 models, 16 models were homo-12-mer oligo-state (Figure 9), and the remaining 7 models included three homodimer and four monomer models (Supplementary Figure 1). The detailed information could be accessed in Supplementary Table 7. For the homo-12-mer models, the different 3D structures were observed in CtHSP20-9, CtHSP20-10, and CtHSP20-30 with low identities of 34.82, 37.86, and 37.86%, respectively (Supplementary Table 7).

**FIGURE 10:**
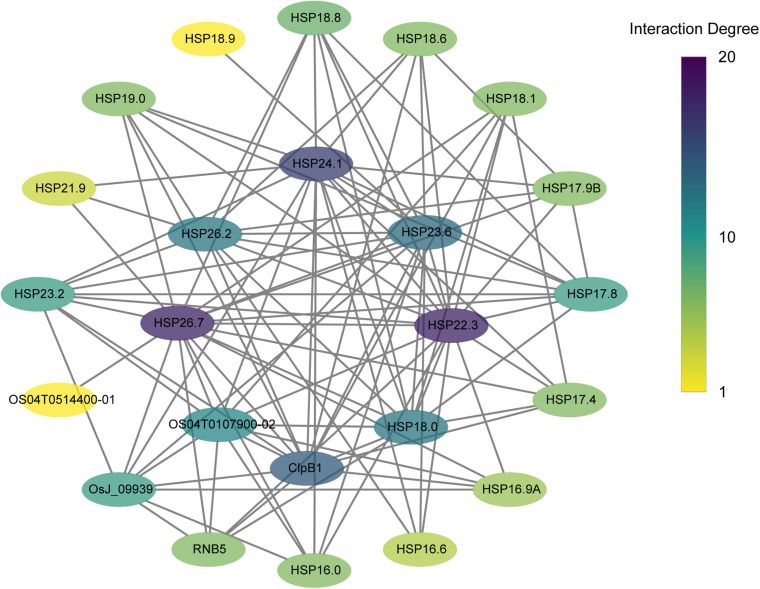
Protein–protein interaction (PPI) networks of *CtHSP20s* and their related proteins. The protein node color represents the interaction degrees linked with each node.

The PPI networks were further analyzed to detect interactions among *CtHSP20s* and related proteins with the STRING website (Damian et al., 2018). Totally, 27 proteins had rice orthologs with identities from 31.4 to 93.4% (Supplementary Table 8). As shown in Figure 10, the proteins interacted with other proteins and might contribute to some biological processes together. The protein nodes were manually rearranged according to interaction degrees. For proteins on the inner circular layout, the interaction degrees were over 10. In addition to HSP20 proteins, ClpB1 (HSP100), OsJ_09939 (HSP70), and Os04T0107900-02 (HSP90) proteins were also highly linked with HSP20 proteins.

## Discussion

Heat shock protein 20s, as molecular chaperones, are a ubiquitous protein family found in both prokaryotes and eukaryotes, and they are the most abundant HSP family in plants (Waters, 2013). With the ever-increasing availability of plant genomes and transcriptomes, the *HSP20* genes have been identified from some monocots and dicots plants, such as *Arabidopsis* (Scharf et al., 2001; Siddique et al., 2008), soybean (Lopes-Caitar et al., 2013), potato (Zhao et al., 2018), apple (Yao et al., 2020), rice (Ouyang et al., 2009), bread wheat (Muthusamy et al., 2017), and switchgrass (Yan et al., 2017). However, no studies have been conducted on an overall identification and characterization of the *HSP20* genes from African bermudagrass, an important warm-season turfgrass species. The completion of high-quality African bermudagrass genome assembly has just provided an opportunity to identify and characterize HSP20s at the whole-genome level (Cui et al., 2021).

Here, a total of 41 *HSP20* genes were identified in African bermudagrass genome. The HSP20 gene number of African bermudagrass was higher than those of *Arabidopsis* (19; Siddique et al., 2008) and rice (39; Ouyang et al., 2009), similar to those of apple (41; Yao et al., 2020) and watermelon (44; He et al., 2018), and less than those of potato (48; Zhao et al., 2018), soybean (51; Lopes-Caitar et al., 2013), and wheat (117; Wang et al., 2017). The differences in HSP20 gene numbers are most likely due to the genome size differences and the fact of gene duplications during plant evolution. Among the species, the number of *HSP20* genes in *Arabidopsis* is the least, which was thought to be related to its smaller genome size (Zhao et al., 2018). Gene duplication plays important roles in the expansion of the number of gene families in plants (Blanc and Wolfe, 2004). The sHSPs are known to undergo a lineage-specific gene expansion, diversifying early in land plant evolution, potentially in response to stress in the terrestrial environment, and expand again in seed plants and again in angiosperms (Wang et al., 2017; Waters and Vierling, 2020). The expansion of *HSP20* genes in African bermudagrass genome was possibly owing to the whole genome duplication events (WGD) during evolution (Cui et al., 2021). The gene duplications were then investigated, and the captured paralogs included six pairs of WGD/segmental duplicates and nine pairs of tandem duplicates. Compared with WGD/segmental duplicates, tandem duplicates accounted for a larger proportion, and they were likely to play more important roles in HSP20 evolution. Additionally, the HSP20 syntenic genes were also predicted in monocots (maize and rice) and dicots (*Arabidopsis* and soybean). As expected, maize and rice contained more syntenic genes with HSP20s of African bermudagrass than *Arabidopsis* and soybean did, which provided the evidence that African bermudagrass had a closer evolutionary relationship with monocot plants than dicot plants (Cui et al., 2021), which indicated that *CtHSP20s* might have experienced species-specific duplications after the monocot/dicot divergence event, and it was also reported that African bermudagrass had experienced two WGD events after the divergence between monocots and dicots (Cui et al., 2021). Only three *CtHSP20s* (CtHSP20-3, CtHSP20-35, and CtHSP20-41) had syntenic genes among four species, and they belonged to the conserved subfamilies CI, Po, and CVI, respectively. Combining the inferred orthologs in Supplementary Table 2, we deducted that these three genes could originate from a common ancestor before the divergence between monocots and dicots, and their functions might be conserved and important in angiosperms.

Furthermore, the phylogenetic tree was utilized to uncover the HSP20s evolutionary relationships. In the current study, 172 HSP20s containing *CtHSP20s* together with HSP20s from other four species were categorized into 12 distinct subfamilies (CI to CVII, MI, MII, ER, P, and Po). Among the sequences, 117 HSP20s accounting for 68.02% were clustered into the cytoplasm subfamilies (CI to CVII), which was consistent with previous findings in other plants (Siddique et al., 2008; Zhao et al., 2018), and the cytoplasm was suggested to be the primary cellular site for HSP20s accumulation and function (Lopes-Caitar et al., 2013; Yao et al., 2020). Interestingly, some HSP20 members clustered in the same subfamily from various species were more related than those in different subfamily belonging to the same species, indicating that there was synteny among different plant species, and these HSP20s were conserved across multiple species (Lopes-Caitar et al., 2013). The colored collinear links in Figure 3A represent the syntenic relationships of HSP20s, and the syntenic genes could evolve from a common ancestor of the HSP20 family. We also noted that M subfamily members were adjacent to P subfamily members, which implied that they might undergo a closer divergence time (Waters and Vierling, 2020). Among *CtHSP20s*, the phylogenetic tree exhibited similar patterns with their motif composition and distribution. There was no *CtHSP20s* in CIV and CVII subfamilies, which might be caused by gene loss during evolution. As for motifs, Motif 1, Motif 2, and Motif 4 were conservative and widespread, and they could be more responsible for HSP20 functions. Overall, the diversity of HSP20 family could be driven by environmental selection pressures and continuous evolution of plants.

*HSP20* genes could be responsible for different stresses (Waters et al., 1996; Yao et al., 2020). In our research, the expression patterns of *CtHSP20s* were investigated on the basis of their transcriptome data from various abiotic stresses. As a result, 41 *CtHSP20s* genes could be classified into three groups (Groups I to III; Figure 6). The unified expression patterns were observed in Group I, and almost all genes were up-regulated under HTS and down-regulated under other treatments. Similarly, many of the HSP20s in various plant species were also up-regulated in response to heat stress (Guo et al., 2015; Zhao et al., 2018; Yao et al., 2020). Considering numerous studies have reported the positive role of sHSPs in plant thermotolerance (Waters, 2013; Haslbeck and Vierling, 2015; Waters and Vierling, 2020), the Group I genes could be the major ones contributing to the heat-stress tolerance, and the functions of these genes needed to be validated in further studies. In Group II, three genes (*CtHSP20-14*, *CtHSP20-35*, and *CtHSP20-36*) maintained higher expression levels under RTS and LTS instead of HTS, and they could be low temperature responsive. Particularly, *CtHSP20-14* showed more obvious down-regulation under HTS, and it was one of the 10 *CtHSP20* genes that had orthologs in the two monocot plants (rice and maize), but not in the two dicot plants (*Arabidopsis* and soybean; Supplementary Table 2). It would be interested to further study the role of *CtHSP20-14* in response to temperature stresses since it could be monocot specific, and its function has not been characterized. The remaining two genes (*CtHSP20-9* and *CtHSP20-10*) only expressed under HTS and LTS treatments. Intriguingly, *CtHSP20-9* and *CtHSP20-10* belonged to the same pair of WGD/segmental duplicates, and experienced positive selection (Ka/Ks > 1) as well. In addition, they were among the most conserved *CtHSP20s* across various plant species according to the orthologous analysis (Supplementary Table 2). Thus, we speculated that CtHSP20-9 and CtHSP20-10 were possibly selected in response to extreme temperatures during evolution. In Group III, almost all the genes were down-regulated among DSS, SSS, HTS and LTS compared with the control, which suggested that these genes could be negatively regulated to adapt to abiotic stresses. Two studies from a research group reported negative effects on growth and stress tolerance in *A. thaliana* plants expressing either an *Agrostis stolonifera* cytosolic AsHSP17 or chloroplast AsHSP26.8a constitutively (Sun et al., 2016, 2020). Due to the importance of *cis*-elements in gene promoters for plant responses to environmental stresses (Yamaguchi-Shinozaki and Shinozaki, 2005), we further identified them in the putative promoter regions of African bermudagrass *HSP20* genes. Based on the results, stress-responsive and hormone-related elements were closely related to abiotic stresses. As reported (Sarkar et al., 2009; Lopes-Caitar et al., 2013), under heat stress, heat shock transcription factors (HSFs) could bind to HSEs to regulate the expression levels of *HSP20* genes. In this study, we further verified that the correlation relationship between HSEs and up-regulated genes was extremely significant (Fisher’s exact test, ^∗∗^*p* < 0.01), which suggested that HSFs could upregulate *HSP20* genes for African bermudagrass to cope with high temperature. Additionally, the light-responsive elements were also widespread among all *CtHSP20s*, which was consistent with the results in apple, grape, and pepper (Guo et al., 2015; Ji et al., 2019; Yao et al., 2020). Light is essential to plant growth and development, and the finding confirmed that HSP20s could be not only important for environmental stresses but also for normal growth and development of plants (Waters and Vierling, 2020; Yao et al., 2020).

Although HSP20s were relatively small among HSP families, HSP20 oligomers often contained 12 to 40 subunits (Basha et al., 2012; Haslbeck and Vierling, 2015), and the ability to assemble into large oligomers of 12 or more subunits is the key to the function of many HSP20s (van Montfort et al., 2001; Hanazono et al., 2013). In our analysis, 16 proteins were homo-12-mer and successful modeling with identities from 34.82 to 83.33%. The structures of CtHSP20-9, CtHSP20-10, and CtHSP20-30 were slightly different from the others (Figure 9) with relatively lower homology modeling identities of 34.82, 37.86, and 37.86%. Combined with the prior analyses, gene duplication and selection events might both contribute to the evolution of CtHSP20-9 and CtHSP20-10. Notably, three HSP20s (CtHSP20-17, CtHSP20-22, and CtHSP20-27) were homodimers based on modeling with identities of 75, 62.37, and 30.77% (Supplementary Figure 1). A few HSP20s were found to be present as small oligomers of two to four subunits (Kokke et al., 1998; Basha et al., 2013). These small oligomers (e.g., tetramers) were thought to be the building blocks of larger HSP20 complexes, and that higher multimer formation was a prerequisite to fulfill their chaperone-like activity (Kokke et al., 1998). A striking feature of HSP20 oligomers is their dynamic behavior. Some HSP20s can readily exchange subunits and form hetero-oligomeric complex in a temperature-dependent manner, which is very likely crucial to their functions (Stengel et al., 2010; Chen et al., 2014). As expected, the integrated PPI networks of *CtHSP20s* found that the majority of the HSP20 proteins were enriched. The interaction degrees of the proteins on the inner circular layout were generally higher than those on the outer ones. In addition to the HSP20 proteins, some ATP-dependent chaperones containing HSP100 (ClpB1), HSP70 (OsJ_039939), and HSP90 (Os04T0107900-02) were also enriched in the networks. The current HSP20/sHSP function model proposes that HSP20s act as ATP-independent molecular chaperones to capture stress-denatured proteins as substrates. The HSP20-bound substrates are then prevented from irreversible denaturation and can be reactivated by ATP-dependent chaperones (HSP70 and co-chaperones, along with the protein disaggregase HSP100) for refolding (Haslbeck and Vierling, 2015). HSP70 and HSP100 could be helpful in releasing and refolding substrate proteins (Haslbeck and Vierling, 2015). In tomato, HSP70 and HSP90 were reported to directly interact with HSFs to regulate downstream gene expressions (Hahn et al., 2011). The PPI networks provided the evidence that different kinds of chaperones could cooperate together in response to environmental stresses, and further researches would be warranted to explore the mechanisms in more detail.

## Conclusion

In this study, a total of 41 *CtHSP20s* were identified and confirmed in African bermudagrass genome. The *CtHSP20s* were randomly localized on different chromosomes, and they were classified into 12 subfamilies based on the phylogenetic tree and cellular locations. The gene duplications, expression profiles, *cis*-elements, 3D structure, and PPI networks were conducted to resolve the characteristics of *CtHSP20s*. The PPI network revealed that different kinds of chaperones could cooperate together in response to environmental stresses. Additionally, the *HSP20* genes was clustered into three groups (Groups I, II, and III) based on the expression profiles, and most *CtHSP20s* were up-regulated under HTS as expected, especially those in Group I. Interestingly, in Group II, a monocot-specific *HSP20*, *CtHSP20-14* maintained higher expression levels under both RTS and LTS, but not HTS. Moreover, a pair of WGD/segmental duplicates *CtHSP20-9* and *CtHSP20-10* were among the most conserved HSP20s across different plant species, and they seemed to be positively selected in response to extreme temperatures during evolution. This work would aid in elucidating further functional characterizations of *CtHSP20s* in the future.

## Data Availability Statement

Publicly available datasets were analyzed in this study. This data can be found here: https://ngdc.cncb.ac.cn/gsub/submit/bioproject/PRJCA003581.

## Author Contributions

FC collected the public dataset, performed the data analysis, and wrote the manuscript. GT helped collect the plant materials. XW and KW conceived this study and reviewed the manuscript. All of the authors read and approved the final manuscript.

## Conflict of Interest

The authors declare that the research was conducted in the absence of any commercial or financial relationships that could be construed as a potential conflict of interest.

## Publisher’s Note

All claims expressed in this article are solely those of the authors and do not necessarily represent those of their affiliated organizations, or those of the publisher, the editors and the reviewers. Any product that may be evaluated in this article, or claim that may be made by its manufacturer, is not guaranteed or endorsed by the publisher.

## Footnotes

1
http://pfam.xfam.org/

2
http://hmmer.org/

3
https://www.ncbi.nlm.nih.gov/Structure/bwrpsb/bwrpsb.cgi

4
http://smart.embl.de/

5
https://web.expasy.org/protparam/

6
http://circos.ca/

7
https://github.com/tanghaibao/jcvi/

8
https://www.megasoftware.net/

9
https://meme-suite.org/meme/tools/meme/

10
https://itol.embl.de/

11
http://bioinformatics.psb.ugent.be/webtools/plantcare/html/

12
https://meme-suite.org/meme/tools/fimo/

13
https://swissmodel.expasy.org/

14
https://string-db.org/

## Supplementary Material

Supplementary Figure 1 The three-dimensional (3D) proteins structures of small oligomers.

Supplementary Table 1 Paralogous gene pairs among *CtHSP20s*.

Supplementary Table 2 The inferred *CtHSP20s* orthologs in *Arabidopsis*, soybean, maize, and rice.

Supplementary Table 3 Ka/Ks values among paralogous gene pairs.

Supplementary Table 4 Ka/Ks values among syntenic gene pairs.

Supplementary Table 5 The identified motif sequences.

Supplementary Table 6 Predicted *cis*-elements results.

Supplementary Table 7 The detailed information of the predicted three-dimensional (3D) proteins.

Supplementary Table 8 The predicted rice orthologs of *CtHSP20s* with STRING.
