# Supplementary figures and images for: Genome-Wide Analysis of the HSP20 Gene Family and Expression Patterns of HSP20 Genes in Response to Abiotic Stresses in Cynodon transvaalensis
Source: Front Genet. 2021 Sep 8;12:732812. doi: 10.3389/fgene.2021.732812 (PMC8455957; doi:10.3389/fgene.2021.732812)

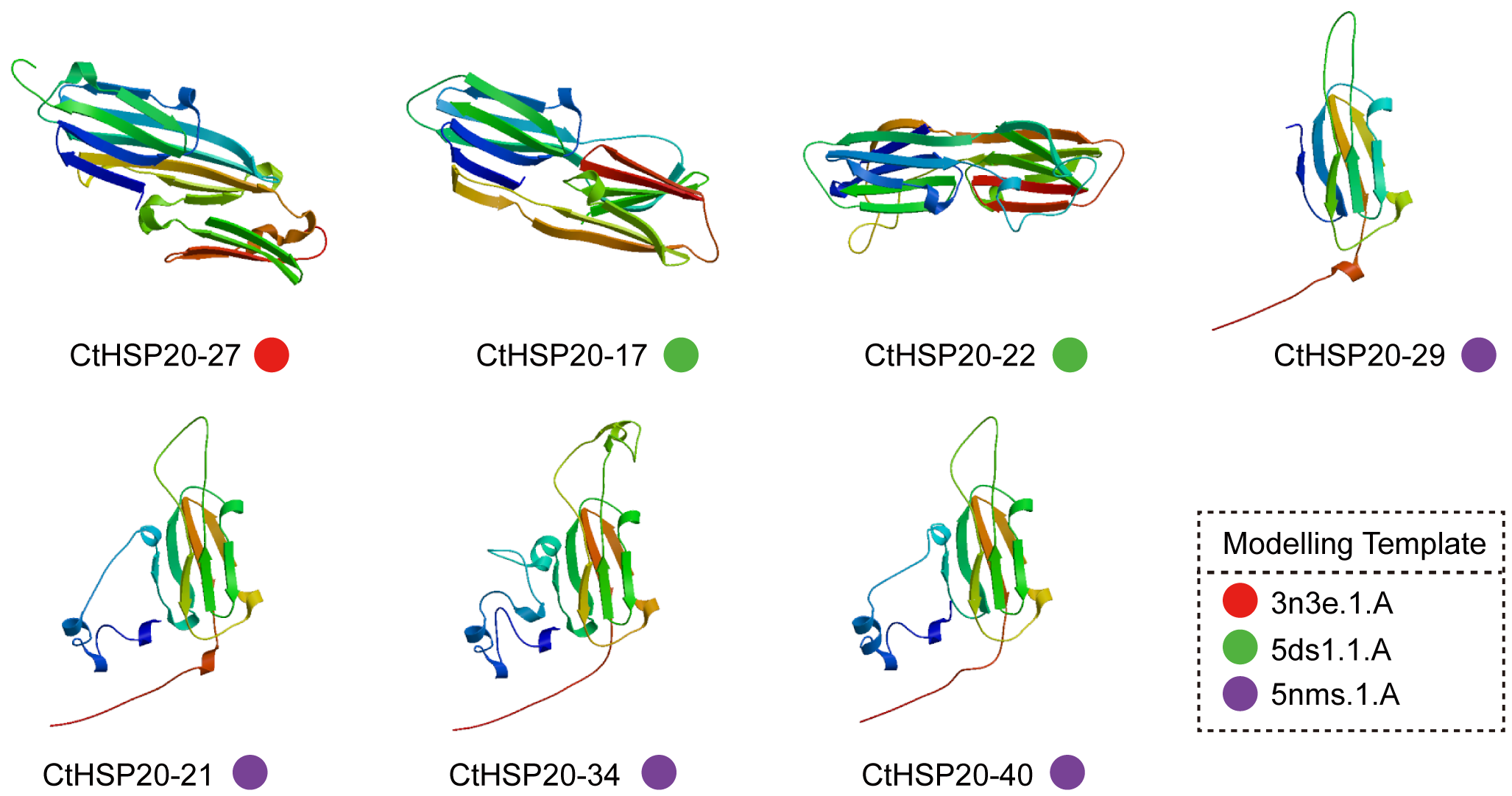

Supplement: Supplementary Figure 1 — The three-dimensional (3D) proteins structures of small oligomers. [file Image_1.TIF]
